# Supplementary material for: A new Devonian euthycarcinoid reveals the use of different respiratory strategies during the marine-to-terrestrial transition in the myriapod lineage
Source: R Soc Open Sci. 2020 Oct 21;7(10):201037. doi: 10.1098/rsos.201037 (PMC7657913; doi:10.1098/rsos.201037)
Supplement: Electronic Supplementary Material [file rsos201037supp1.docx]

**Electronic Supplementary Material for manuscript:**

**A new Devonian euthycarcinoid reveals the use of different respiratory strategies during the marine-to-terrestrial transition in the myriapod lineage**

Pierre Gueriau^1,2^, James C. Lamsdell^3^, Roy A. Wogelius^4^, Phillip L. Manning^4,5^, Victoria M. Egerton^4,5^, Uwe Bergmann^6^, Loïc Bertrand^1,7^ and Julien Denayer^8^

^1^ Université Paris-Saclay, CNRS, ministère de la Culture, UVSQ, MNHN, Institut photonique d'analyse non-destructive européen des matériaux anciens, 91192, Saint-Aubin, France.

^2^ Institute of Earth Sciences, University of Lausanne, Géopolis, CH-1015 Lausanne, Switzerland.

^3^ Department of Geology and Geography, West Virginia University, 98 Beechurst Avenue, Morgantown, WV 26505, USA.

^4^ University of Manchester, Interdisciplinary Centre for Ancient Life, Department of Earth and Environmental Sciences, University of Manchester, Manchester M13 9PL, UK.

^5^ The Children’s Museum of Indianapolis, 3000 N Meridian St, Indianapolis, IN 46208, USA.

^6^ Stanford PULSE Institute, SLAC National Accelerator Laboratory, Menlo Park, CA 94025, USA.

^7^ Université Paris-Saclay, 91190 Saint-Aubin, France

^8^ Evolution and Diversity Dynamics Lab, Geology Research Unit, University of Liège, Allée du Six‐Août, B18, Sart Tilman, B4000 Liège, Belgium.

*Author for correspondence: Pierre Gueriau; email: pierre.gueriau@hotmail.fr

ORCID

PG, 0000-0002-7529-3456

JCL, 0000-0002-1045-9574

RAW, 0000-0002-5781-2152

PLM, 0000-0002-7161-6246

VME, 0000-0002-0739-6533

UB, 0000-0001-5639-166X

LB, 0000-0001-6622-9113

JD, 0000-0002-4339-7760

**Geological background**

The specimen described herein comes from the Pont de Bonne locality. This locality is located in the central part of the Dinant Synclinorium (figure 1), which was part of the Namur–Dinant Basin situated along the southeastern margin of Laurussia during the Devonian and Carboniferous. It is a classic locality of the Hoyoux valley for the study of the Upper Devonian, and particularly for the Famennian proximal siliciclastic succession (e.g. [S1]). It is also famous for its plant fossils [34] and euryterid remains [35]. The Upper Famennian Evieux Formation crops out in small disused quarries situated along the Liège-Ciney road, north of the bridge upon the Hoyoux. Fraipont [35] gave a complete description of the section that is nowadays partly inaccessible and walled. From north to south, he described a 3.5 m-thick unit of micaceous sandstone (unit A in [35], quarried), 3 m of micaceous sandstone, partly calcareous with some fish remains (unit B), 4 m of dolomitic micaceous sandstone (unit C, quarried), 6 m of sandstone and siltstone with some fish remains (unit D), 5.5 m of sandstone-shale alternation (units E–J). The eurypterids described by Fraipont [35] and Størmer & Waterston [36] come from the greenish shales of unit E. This 1.5 m-thick shaly layer is unfortunately not accessible anymore and recent research failed to recover Fraipont’s [35] fossiliferous layer.

During Late Famennian times, the Namur-Dinant basin recorded current-dominated tidal flat deposits on which a sandy barrier acted as a barrier. The back-barrier inter- to supratidal lagoon received sediments both from offshore (tide and currents) and from the fluvial discharge [37,S2,S3]. At Pont de Bonne, the Evieux Formation witnesses tidal flat and lagoonal settings [37]. The later are locally developed as slightly dolomitic shale with *Racophyton* remains. It is commonly acknowledged that this fern grew in brackish water marshes [38]. The euthycarcinoid specimen described herein was collected in 1978 in such a *Racophyton* dolomitic shale in the top of unit B. Contrary to Fraipont’s material, this new specimen is not disarticulated, indicating (most likely) very little post-mortem (or post-moulting) transport. It also presents an uncommon limonitic preservation, similar to that of plant remains coming from the same bed.

**References**

1. Stockmans, F. 1948. Végétaux du Dévonien supérieur de la Belgique. *Mémoires du Musée royal d'histoire naturelle de Belgique* 110, 1–85.
2. Fraipont, J. 1889. Euryptérides nouveaux du Dévonien supérieur de Belgique (Psammites du Condroz). *Annales de la Société géologique de Belgique* 17, 53–62.
3. Størmer, L. & Waterston, C.D., 1968. *Cyrtoctenus* gen. nov., a large Late Palaeozoic arthropod with pectinate appendages. *Transaction of the Royal Society of Edinburgh* 68, 1–104.
4. Thorez, J, & Dreesen, R. 1986. A model of a regressive depositional system around the Old Red Continent as exemplified by a field trip in the Upper Famennian ‘Psammites du Condroz’ in Belgium. *Annales de la Société géologique de Belgique* 109, 285–323.
5. Scheckler, S.E. 1986. Geology, floristics and paleoecology of Late Devonian coal swamps from Appalachian Laurentia (U.S.A.). Annales de la Société géologique de Belgique 109, 209–222.
6. Thorez, J. 1964. Sédimentation rhytmique du Famennien supérieur dans la vallée du Hoyoux (Bassin de Dinant, Belgique). *Annales de la Société géologique de Belgique* 87, 1–51.
7. Thorez, J., Streel, M., Bouckaert, J. & Bless, M. 1977. Stratigraphie et paléogéographie de la partie orientale du Synclinorium de Dinant (Belgique) au Famennien supérieur: un modèle de bassin sédimentaire reconstitué par analyse pluridisciplinaire sédimentologique et micropaléontologique. *Mededelingen rijks geologische dienst* 28, 17–32.
8. Denayer, J., Prestianni, C., Gueriau, P., Olive, S. & Clément G. 2016. Stratigraphy and depositional environments of the Late Famennian (Late Devonian) of Southern Belgium and characterization of the Strud locality. *Geological Magazine* 153, 128–142.

**
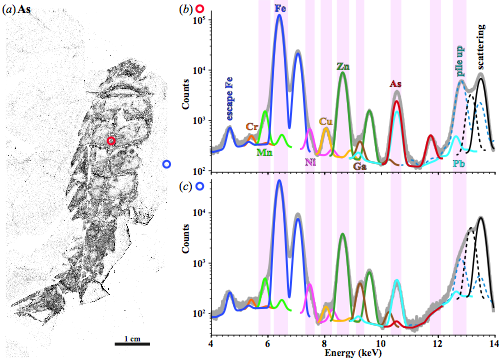
**

**Figure S1.** XRF spectra from *Ericixerxes* *potii* gen. et sp. nov. and its sedimentary matrix. (*a*) SRS-XRF elemental maps of As Kα_1_ emission line, showing the localisation of full XRF spectra collected using a count time of 30 s. (*b, c*) XRF spectra and main elemental contributions (obtained from fitting using the PyMCA data-analysis software [40]) from the cuticle (*b*) and matrix (*c*). Pink areas highlight the preselected spectral regions used to extract integrated intensities of elements.

**
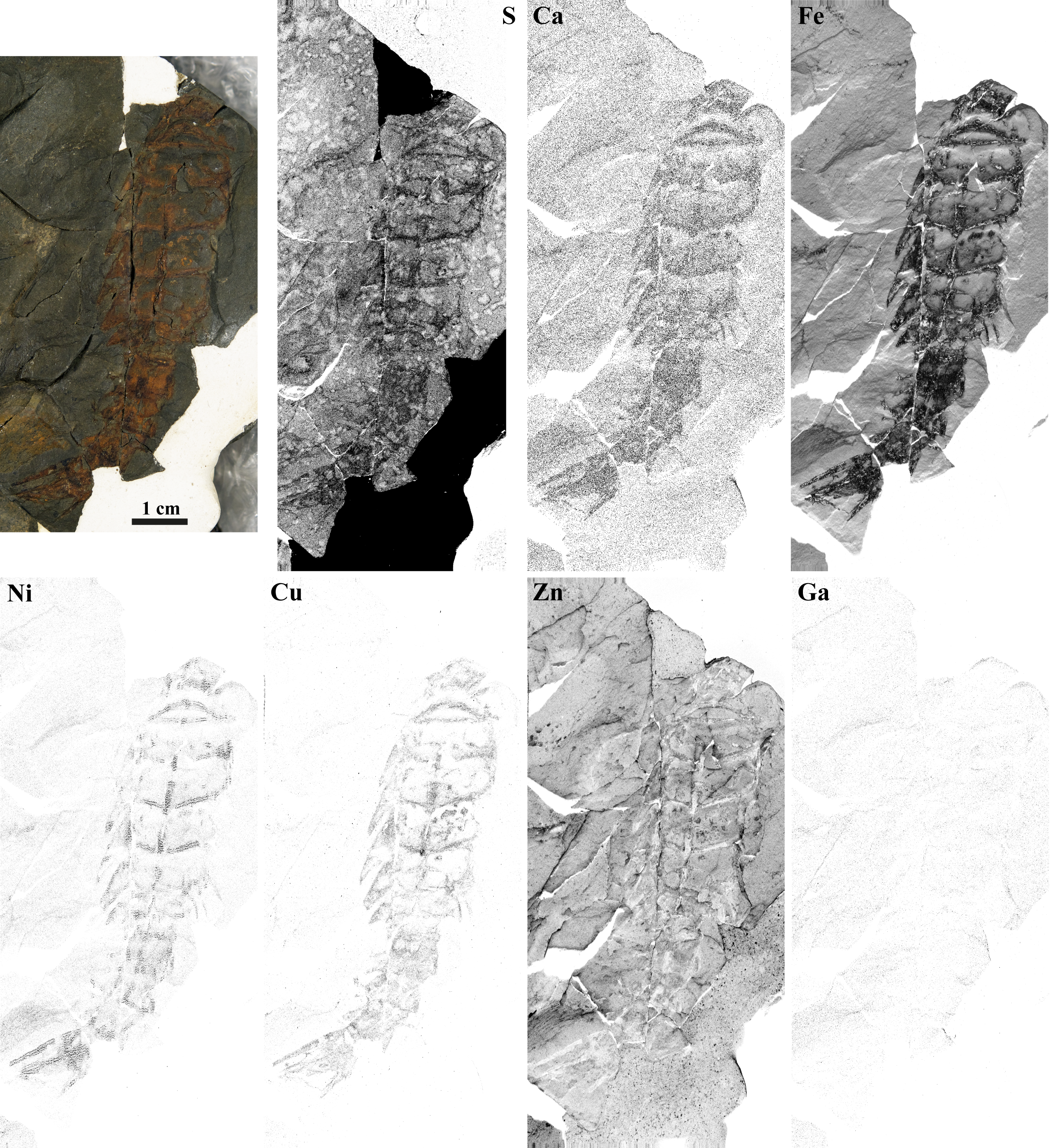
**

**Figure S2.** Optical photograph of *Ericixerxes* *potii* gen. et sp. nov., and additional SRS-XRF elemental distributions of S (Kα_1_ emission line) mapped using an incident beam energy of 3.15 keV, and of Ca, Fe, Ni, Cu, Zn, Ga (Kα_1_ lines) mapped using an incident beam energy of 13.5 keV. Mapping parameters: scanning step: 50 µm; 801×2001 pixels for S, 901×2001 pixels for the other elements; colour scale goes from white (low abundance) to black (high abundance).
